# Sale of Food and Drugs Bill

**Published:** 1899-07-29

**Authors:** 


### The Hospital

A Journal of

The Medical Sciences and Hospital Administration.

Vol. XXVI.—No. 670.] Edited by Sir Henry Burdett, K.C.B., and Solomon C. Smith, M.D., M.R.C.P. [July 29, 1899.

### Sale of Food and Drugs Bill.

Under whatever form the Sale of Food and Drugs Bill may ultimately be placed upon the Statute Book, there will at least be no cause to complain of it as a piece of hasty legislation, or to assert that it failed to receive from the House of Commons the attention due to its importance. After the second reading, and after being referred to and amended by a Grand Committee, it still occupied the House on four nights of last week, at the close of which the consideration of its provisions was once more adjourned, and the third reading was not carried until Monday. There was, indeed, what can hardly be described in any other way than as a deliberate attempt to wreck the Bill, or, failing that, to deprive it of many of its most valuable provisions; and, although the dissentients were beaten in every division by majorities of about four or five to one, their pertinacity was in no way diminished by defeat. The more important discussions turned upon three main points—the substitution of margarine for butter, the properly protective labelling of impoverished milk so that its worthlessness as a food for infants may be declared, and the infliction of imprisonment in lieu of fine for repeated breaches of the law. It is a matter of common knowledge, and has recently been abundantly shown by a large number of successful prosecutions, that margarine is extensively sold under the name of butter, and that in this way purchasers, chiefly among the industrial classes, are defrauded to an enormous extent, not so much in the nutritive value of what is sold to them as in respect of the price which they are made to pay for it. Roughly speaking, the proper price of margarine is about half the proper price of butter, so that when it is sold under the name of butter the purchaser pays for his commodity about twice as much as he ought to do. Nominally for the protection of the purchaser, but really on quite a different ground, the Government was urged to introduce into the measure a prohibition of the use of colouring matter for margarine, it being alleged that it ought to be left in its natural white condition, or that, if coloured at all, the colouring matter should be pink, or blue, or green, or anything but one of the various tints of yellow which we are accustomed to see in different samples of genuine butter. It was successfully contended in reply that the purchaser of margarine was entitled to obtain the gratification of his eye as well as that of his palate, and that there could be no valid objection, as long as he was not cheated as to the nature of his purchase, to giving it any sort of appearance by which the demand for it might be promoted. A provision of the Bill which occasioned much discussion was that to enact that margarine must not contain more than 10 per cent. of butter fat. It appears that a certain quantity of milk is used in the process of manufacture, and that, on this account, a certain admixture of butter fat, usually amounting to about 5 per cent., is unavoidable, but that if the admixture amounted to more than 10 per cent. the difficulties of the analyst would become very great, while the nutritive value of the compound would not really be enhanced. The discussions and divisions on these points, however, amounted to little more than endeavours on the part of some members to defend the agricultural interest as against the purveyors of margarine, and, on the part of others, to protect manufacturers against legislation which might, it was thought, unduly hamper their freedom. When the question of impoverished milk was reached, it was satisfactory to see that these considerations were no longer prominent, and that the amendments urged were almost entirely in the direction of protecting infants against disease and mortality incidental to the use of innutritious food. Various suggestions were made with regard to the nature of the warnings which the seller should be bound to affix to every tin containing impoverished milk, and the chief difficulty seemed to be to find room for the conspicuous printing of all that it might be desirable to say. Mr. Bartley made the terse suggestion that the label should bear the words "Bad for babies," and finally Mr. Long undertook that his department would do their best to devise a suitable inscription. The remaining question, that of the imprisonment of offenders, raised a considerable outcry, and some honourable members drew piteous pictures of the honest, but careless, tradesman who, solely by the fault of his assistant or servant, found himself sentenced to undergo three months' incarceration. But these pictures drew no tears, and Mr. Long had an easy task in showing that they were not likely to be realised in actual life. The penalty of imprisonment will only be incurred upon a third conviction, and then at the discretion of the magistrate, who will retain the power to inflict a fine if he thinks such a course called for by the circumstances. It is impossible to read the newspapers without being convinced that fines of a few pounds at a time are not sufficient to deter certain tradesmen from the habitual commission of profitable fraud, and that the further powers asked for by the Government will be of great value for the protection of the consumer.

